# Cryogel System Based on Poly(vinyl alcohol)/Poly(ethylene brassylate-co-squaric acid) Platform with Dual Bioactive Activity

**DOI:** 10.3390/gels9030174

**Published:** 2023-02-22

**Authors:** Bianca-Elena-Beatrice Crețu, Alina Gabriela Rusu, Alina Ghilan, Irina Rosca, Loredana Elena Nita, Aurica P. Chiriac

**Affiliations:** 1 Department of Natural Polymers, Bioactive and Biocompatible Materials, Petru Poni Institute of Macromolecular Chemistry, 41 A Grigore Ghica Voda Alley, 700487 Iasi, Romania; 2 Center of Advanced Research in Bionanoconjugates and Biopolymers, Petru Poni Institute of Macromolecular Chemistry, 41 A Grigore Ghica Voda Alley, 700487 Iasi, Romania

**Keywords:** poly(vinyl alcohol), copolymacrolactone, thymol, α-tocopherol, synergistic effect, hydrogel dressings

## Abstract

The inability to meet and ensure as many requirements as possible is fully justified by the continuous interest in obtaining new multifunctional materials. A new cryogel system based on poly(vinyl alcohol) (PVA) and poly(ethylene brassylate-co-squaric acid) (PEBSA) obtained by repeated freeze–thaw processes was previously reported and used for the incorporation of an antibacterial essential oil—namely, thymol (Thy). Furthermore, the present study aims to confer antioxidant properties to the PVA/PEBSA_Thy system by encapsulating α-tocopherol (α-Tcp), targeting a double therapeutic effect due to the presence of both bioactive compounds. The amphiphilic nature of the PEBSA copolymer allowed for the encapsulation of both Thy and α-Tcp, via an in situ entrapment method. The new PVA/PEBSA_Thy_α-Tcp systems were characterized in terms of their influence on the composition, network morphology and release profiles, as well as their antimicrobial and antioxidant properties. The study underlined the cumulative antioxidant efficiency of Thy and α-Tcp, which in combination with the PEBSA copolymer have a synergistic effect (97.1%). We believe that the convenient and simple strategy offered in this study increases applicability for these new PVA/PEBSA_Thy_α-Tcp cryogel systems.

## 1. Introduction

A wound is defined as the disruption of the anatomical structure and normal function of the skin, caused by various types of trauma, burns, or surgery [1]. As the wound occurs, the healing process begins [2]. During this process, the generation of an excess of reactive oxygen species (ROS) also occurs as part of the defense mechanism against pathogens. ROSs are small molecules derived from unstable oxygen that try to stabilize themselves by capturing the electrons of some molecules in living organisms, implying the appearance of associated complications (dysfunctions at the level of cell membranes, conformational changes in proteins, loss of enzyme roles along with breaking DNA chains) [3,4].

Antioxidants are chemical compounds that can alleviate oxidative stress by donating electrons to other molecules, such as ROSs, and support the wound healing process [5]. Antioxidants can be classified as endogenous, produced naturally in the body as, for example, superoxide dismutase (SOD), catalase (CAT), glutathione peroxidase (GPx), and exogenous obtained through the diet, such as carotenoids, vitamin C, vitamin E and flavonoids, among others [6]. Among the tocopherols, α-Tcp is the most abundant form of vitamin E and is considered a powerful fat-soluble antioxidant that protects membrane lipids against oxidation and contributes to the mechanical stabilization of membranes through Van der Waals physical interactions [7]. The administration of α-tocopherol, as well as the other liposoluble vitamins, presents some particularly important challenges due to its low water solubility and stability [8]. Moreover, vitamins are sensitive molecules so they require protection from pro-oxidant factors such as oxygen, UV or high temperatures. In this sense, the encapsulation of vitamins into a polymeric network could be a promising approach to preserve their chemical integrity and effectiveness, but also their controlled release, thus reducing the occurrence of hypervitaminosis syndrome [9]. Conventional hydrogels are commonly used by scientists to overcome the problems mentioned above, but the problems surrounding their implementation on a large scale increase due to the closed and small cavity of hydrogels. Cryogels have received tremendous attention in applications targeting the controlled release of active principles and tissue engineering of the skin, considering their large pore size, rough surface, absorption capacity and rapid swelling [10,11]. PVA is one of the most investigated water-soluble synthetic polymers in obtaining cryogels, with applications as drug carriers, in wound dressings and for tissue engineering due to its biocompatibility, biodegradability and non-toxicity [12].

A new cryogel system based on PVA and PEBSA obtained by repeated freeze–thaw processes was previously reported and used for the incorporation of an antibacterial essential oil—namely, Thy [13]. Thy, chemically known as 2-isopropyl-5-methylphenol, is a natural monoterpenoid phenol which is isolated from *Thymus vulgaris* and other plants such as *Ocimum gratissimum* L., *Origanum* L., *Satureja thymbra* L. [14]. Various pharmacological properties of thymol have been investigated and reported, including antimicrobial, antifungal, antioxidant, anti-inflammatory, analgesic, and healing activities [15,16,17,18]. PEBSA, a copolymacrolactone system was synthetized from ethylene brassylate (EB) and squaric acid (SA) by the ring-opening copolymerization procedure described before [19,20]. The supramolecular structure and high functionality of PEBSA copolymer, as well as its biocompatibility and good thermal stability, have led to it being recommended as a matrix for the incorporation of hydrophobic bioactive compounds [21,22].

In order to achieve a system with dual effect and activity, specifically antioxidant and antimicrobial, the alfa-tocopherol-thymol bioactive formulation was encapsulated in the new PVA/PEBSA polymer network. The PEBSA copolymer, due to its amphiphilic character, allowed for the encapsulation of hydrophobic bioactive substances such us Thy and α-Tcp, via an entrapment in situ method. Moreover, the hydrophobic affinity of the compounds involved in the system led to a good dispersion of the bioactive molecular agents in the PEBSA polymer network [20]. The newly prepared bioactive complexes were characterized in terms of the influence of the composition on the network morphology and release profiles, as well as the dual bioactive behavior due to the presence of Thy and α-Tcp. To support previous studies, PVA/PEBSA_Thy_α-Tcp systems were prepared using PEBSA with three different ratios between EB and SA comonomers, namely, 25/75, 50/50 and 75/25. To our knowledge, there have been no reported studies focusing on the synergistic effect between the antimicrobial properties of Thy and the antioxidant properties of α-Tcp associated with biomedical applications. Overall, this study offers a convenient strategy to achieve a PVA/PEBSA cryogel system with dual therapeutic effect due to the presence of both bioactive compounds. We intend to use the PVA/PEBSA_Thy_α-Tcp cryogel system for antimicrobial and wound healing applications, and in this regard, we carry out the necessary studies.

## 2. Results and Discussion

**Figure 1:**
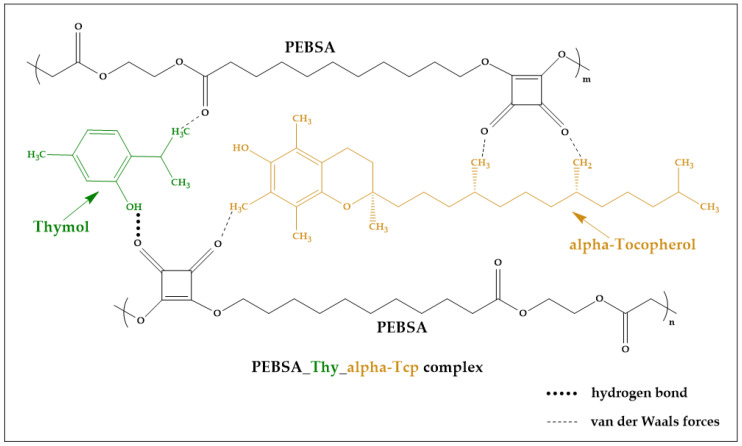
Illustration of PEBSA_Thy_α-Tcp bioactive complex formation.

The present study aims to obtain cryogels based on PVA/PEBSA incorporating Thy and α-Tcp, targeting dual activity generated by the presence of the two bioactive compounds. Our group envisioned this approach to enhance the efficacy of these new antimicrobial cryogels to be used as wound dressings. The encapsulation of the bioactive compounds into the PEBSA polymeric matrix was realized by an inclusion complexation performed in 1,4-dioxane by entrapping of Thy, of α-Tcp, into the amphiphilic PEBSA network (Figure 1).

### 2.1. Morphological Analysis

**Figure 2:**
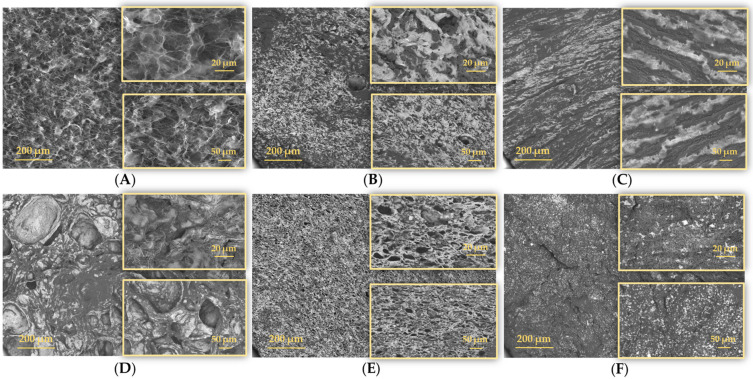
Comparative SEM images of the cryogels: (**A**) PVA_PEBSA_50/50_, (**B**) PVA_PEBSA_25/75__Thy_α-Tcp, (**C**) PVA_PEBSA_50/50__Thy_α-Tcp, (**D**) PVA_PEBSA_75/25__Thy_α-Tcp, (**E**) PVA_PEBSA_50/50__2xThy_α-Tcp, and (**F**) PVA_PEBSA_50/50__Thy_2xα-Tcp.

The internal morphology of the PVA/PEBSA polymer matrix and PVA/PEBSA_Thy_α-Tcp systems were evaluated by SEM. As can be seen in Figure 2A, the SEM micrographs of cryogels based on PVA and PEBSA copolymacrolactone illustrate their three-dimensional network with interconnected honeycomb-like pores and numerous meshes, which can ensure the incorporation of bioactive molecular compounds. The morphology of the PVA/PEBSA_Thy_α-Tcp systems highlights a relatively uniform distribution of the bioactive substances on the surface of the polymer network. The homogeneous distribution of Thy and α-Tcp in the PEBSA polymer network is due to the amphiphilic character of the copolymer and also to the hydrophobic affinity of the compounds involved in the system. Some differences in their morphology result from the cryogel preparation condition namely, the ratio between EB and SA comonomers in the copolymacrolactone system as well as the amount of bioactive compound.

Therefore, in the case of the PVA_PEBSA_Thy_α-Tcp sample (Figure 2B–D), with the increase in the EB/SA ratio in the sample, the pore size increases. This is associated with an increase in the number of carbonyl functional groups in SA, which further results in a higher number of hydrogen bonds between the mixing partners. Thus, the structure and pore size of PVA/PEBSA cryogels can be modified by adjusting the ratio between EB and SA comonomers from PEBSA composition. The morphological characterization of the new variants of cryogels is consistent with that recently reported in the literature [13].

### 2.2. Release Studies

**Figure 3:**
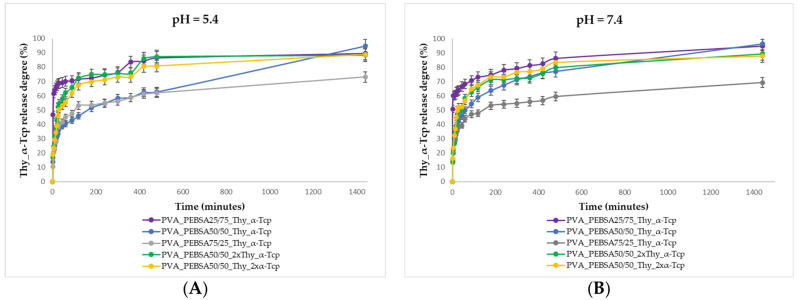
Cumulative release of Thy and α-Tcp at (**A**) pH 5.4 compared to (**B**) pH 7.4. Graphical data are expressed as mean ± standard error of the mean.

The burst release of drugs from therapeutic formulations is not desirable, especially in the treatment of wound infections [23]. Therefore, incorporating these active principles into an effective delivery system, such as a hydrogel, and releasing them in a controlled manner reduces potential side effects. In the reported literature, there are studies that focused on the controlled release of Thy and α-Tcp from a hydrogel polymer matrix—examples are shown in Table 1:

Starting from the premise that pH 5.4 is representative of healthy skin which can range between 5.4 and 8 when the deep layers of the skin are exposed following injuries [31], in this study, the cumulative release of the bioactive compounds was investigated at two different pHs: 5.4 and 7.4 (Figure 3).

The initial burst release of Thy_α-Tcp complex from PVA_PEBSA_25/75__Thy_α-Tcp cryogel, 68.71% at pH 5.4 and 64% at pH 7.4, was observed in the first 30 min, while only 39.71% (pH 5.4) and 39.23% (pH 7.4) of bioactive compounds was released from PVA_PEBSA_75/25__Thy_α-Tcp cryogel at the same time. The release of a smaller amount of the bioactive substance during the burst release step in the case of the PVA_PEBSA_75/25__Thy_α-Tcp system correlates with a stronger hydrophobic character of PVA_PEBSA_75/25_ matrix due to the higher ratio of EB. Therefore, the SA carbonyl groups ensure the coupling of bioactive compounds in the polymer matrix, while the hydrophobic alkyl chains of EB constitute the shell of the complex. Consequently, increasing the amount of EB comonomer to 75% in the chemical structure of the copolymer will determine the immobilization of the bioactive compounds in the network and their controlled release in a pulsating regime.

In order to study the influence of Thy_α-Tcp complex loading in the polymer matrix on the release capacity of the new bioactive compounds, the system with the equimolecular ratio between monomers was selected to vary the amount of bioactive compound (PEBSA_50/50__2xThy_α-Tcp and PEBSA_50/50__Thy_2xα-Tcp). The PVA_PEBSA_50/50__2xThy_α-Tcp and PVA_PEBSA_50/50__Thy_2xα-Tcp systems present a “burst” release of Thy and α-Tcp attributed to the diffusion of a double amount of bioactive compounds. According to Figure 3, the minimum and maximum release rates of Thy and α-Tcp from the studied systems over 24 h were 94.83% (pH 7.4) for the PVA_PEBSA_25/75__Thy_α-Tcp sample and 69.22% (pH 5.4) in the case of the PVA_PEBSA_75/25__Thy_α-Tcp sample. Since the largest number of granulocytes appear after 12–24 h at the injury site, the cells responsible for the immune response against microbial agents, the risk of infection is higher in the first minutes and hours after wounding. Consequently, the first 24 h after the appearance of the injury is the most important time interval to intervene with a material with antimicrobial properties to prevent infection. Therefore, the rate of drug release from hydrogel dressings is a significant factor in preventing infection [32].

### 2.3. Antimicrobial Activity

**Figure 4:**
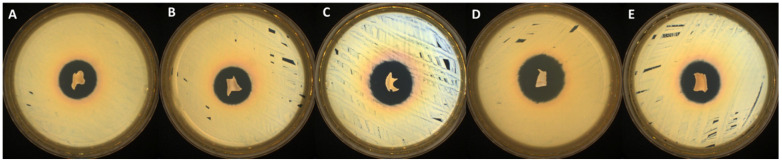
Antibacterial activity of the tested samples: (**A**) PVA_PEBSA_25/75__Thy_α-Tcp, (**B**) PVA_PEBSA_50/50__Thy_α-Tcp, (**C**) PVA_PEBSA_75/25__Thy_α-Tcp, (**D**) PVA_PEBSA_50/50__2xThy_α-Tcp, and (**E**) PVA_PEBSA_50/50__Thy_2xα-Tcp against *S. aureus*.

**Figure 5:**
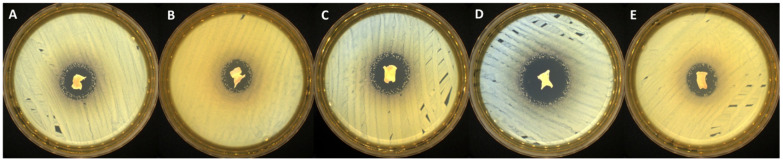
Antibacterial activity of the tested samples: (**A**) PVA_PEBSA_25/75__Thy_α-Tcp, (**B**) PVA_PEBSA_50/50__Thy_α-Tcp, (**C**) PVA_PEBSA_75/25__Thy_α-Tcp, (**D**) PVA_PEBSA_50/50__2xThy_α-Tcp, and (**E**) PVA_PEBSA_50/50__Thy_2xα-Tcp against *E. coli*.

**Figure 6:**
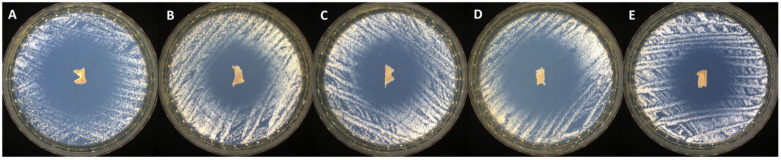
Antifungal activity of the tested samples: (**A**) PVA_PEBSA_25/75__Thy_α-Tcp, (**B**) PVA_PEBSA_50/50__Thy_α-Tcp, (**C**) PVA_PEBSA_75/25__Thy_α-Tcp, (**D**) PVA_PEBSA_50/50__2xThy_α-Tcp, and (**E**) PVA_PEBSA_50/50__Thy_2xα-Tcp against *C. albicans*.

The antimicrobial activity screening of the newly synthesized bioactive compounds against *S. aureus* (Gram-positive bacterial strain), *C. albicans* (fungal strain), and *E. coli* (Gram-negative bacterial strain) was determined by disk diffusion assay. All the tested samples presented antimicrobial activity against the selected reference strains (as presented in Table 2 and Figure 4, Figure 5 and Figure 6), results that correlate very well with recently reported antimicrobial assays [13].

The samples proved to be very effective especially against fungal strain represented by *C. albicans* (up to 38 mm of inhibition zone). Moreover, no significant differences were noticed in terms of antimicrobial activity among systems with different ratios between EB/SA comonomers. A smaller zone of inhibition was noticed in case of PVA_PEBSA_50/50__Thy_2xα-Tcp system against *E. coli* (19 mm), while the PVA_PEBSA_50/50__2xThy_α-Tcp system was more effective against all the tested microbial strains (>27 mm of inhibition zone)—Table 2, the efficiency related to the presence of Thy in a higher ratio. Compared to previous results [13], the addition of α-Tcp to the PVA_72000__PEBSA_Thy system did not substantially affect the antimicrobial character of the samples.

### 2.4. Antioxidant Efficiency

**Figure 7:**
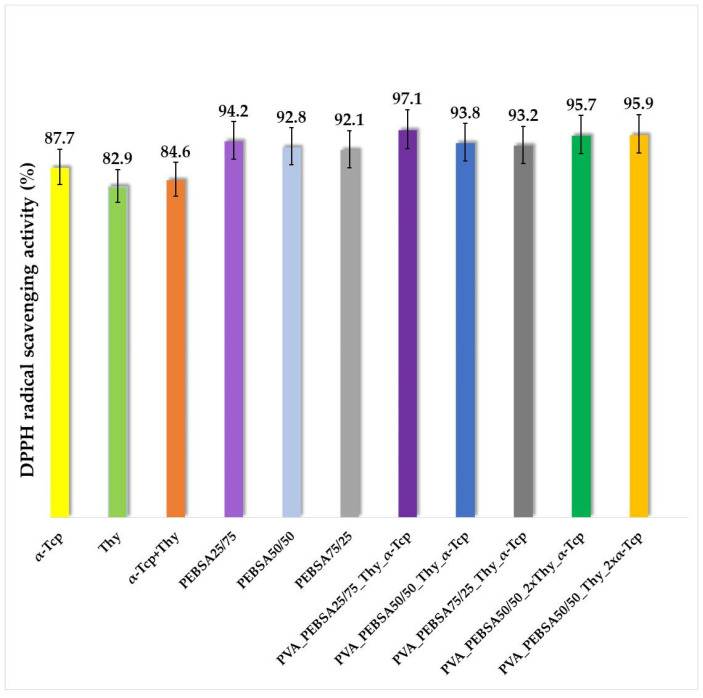
The antioxidant activity of the positive controls and PVA/PEBSA_Thy_α-Tcp systems. Graphical data are expressed as mean ± standard error of the mean.

The interest of researchers in identifying new combinations of biomaterials that exhibit antimicrobial, antioxidant, anti-inflammatory, and healing activities for the treatment of wounds as well as their associated complications is increasing. α-Tcp, the most abundant form of vitamin E, is well known for its strong endogenous antioxidant activity by protecting membrane lipids against oxidation and mechanically stabilizing membranes, improving wound healing and skin regeneration [25]. Supplementing the PVA/PEBSA_Thy system with this antioxidant molecule targets a dual therapy and effect with these two bioactive compounds. The DPPH radical scavenging activity of the synthesized bioactive compounds is illustrated in Figure 7.

According to the data obtained in this study, all samples showed antioxidant activity. The most evident activity is observed in the case of the PVA_PEBSA_25/75__Thy_α-Tcp system (97.1%) containing the PEBSA_25/75_ variant. The carbonyl groups in SA have the ability to accept hydrogen; therefore, increasing the content of SA in the matrix determines a better free radical scavenging activity. At the same time, the cumulative antioxidant efficiency of Thy and α-Tcp in combination with the PEBSA copolymer has a remarkable synergistic effect, between 93.2% and 97.1% (Figure 7). In summary, the mixture of the two bioactive compounds and PEBSA produces new cryogels with potential applications as wound dressings whose therapeutic effects are superior to the effects produced by each individual component.

## 3. Conclusions

The encapsulation of hydrophobic molecular compounds into a polymer matrix has emerged as a method to modulate the low solubility in water and a promising approach to preserve their chemical integrity, efficacy, but also their controlled release in a pulsating or continuous regime. Three-dimensional scaffolds based on cryogels are strong candidates and of particular interest for this purpose [33]. In this study, our group used this strategy to develop new cryogels with antimicrobial and antioxidant activity based on PVA, PEBSA, Thy, and α-Tcp obtained by a repeated freeze–thaw process. On the one hand, Thy shows antimicrobial properties on a wide spectrum of bacteria (e.g., *S. aureus*, *Bacillus licheniformis*, *E. coli*, *P. vulgaris, C. albicans*, etc.) [16] and on the other hand, the encapsulation of α-Tcp confers antioxidant properties to the PVA/PEBSA_Thy system, targeting a double therapeutic effect due to the presence of both bioactive molecular agents. The new bioactive compounds prepared by encapsulation of Thy and α-Tcp into the PVA/PEBSA system were investigated from the point of view of the influence of the composition on the network morphology, release profiles, antimicrobial and antioxidant dual activity. SEM micrographs of the PVA/PEBSA polymer matrix illustrated their three-dimensional structure with interconnected pores and numerous meshes, which can ensure the incorporation of small molecular compounds. The morphology of PVA/PEBSA_Thy_α-Tcp systems highlights a relatively uniform distribution of the bioactive substances on the surface of the polymer network. The homogeneous distribution of Thy and α-Tcp into the PEBSA polymer network is due to the amphiphilic character of the copolymer and also to the hydrophobic affinity of the compounds involved in the system. Thy_α-Tcp release profiles from polymeric cryogels confirm the ability of PVA/PEBSA system to encapsulate these bioactive compounds. The lower release rate of Thy and α-Tcp during the burst release step in the case of the PVA_PEBSA_75/25__Thy_α-Tcp system (39.71% at pH 5.4 and 39.23% at pH 7.4) correlates with a stronger hydrophobic character of the PVA_PEBSA_75/25_ matrix (due to a higher ratio of EB), determining the immobilization of the bioactive compounds in the network and their controlled release. The new PVA/PEBSA_Thy_α-Tcp systems proved antimicrobial activity against *S. aureus* (Gram-positive bacterial strain), *C. albicans* (fungal strain), and *E. coli* (Gram-negative bacterial strain). The study also underlined the cumulative antioxidant efficiency of Thy and α-Tcp, which in combination with the PEBSA copolymer have a synergistic effect (97.1%). However, this high potential of the investigated systems needs to be extensively evaluated by additional studies, in vitro and in vivo, with focus on possible cytotoxicity concerns, as well as their applicability in the management of skin wounds. The design of multifunctional hydrogel dressings with good adaptability to wounds and with painless on-demand removal property to avoid bacterial colonization remains a major problem to be solved [34,35].

## 4. Materials and Methods

### 4.1. Materials

Ethylene brassylate (EB, 1,4-dioxacycloheptadecane-5,17-dione, C_15_H_26_O_4_, M_w_ = 270.36 g/mol, purity of 95.0%), squaric acid (SA, 3,4-dihydroxy-3-cyclobutene-1,2-dione, H_2_C_4_O_4_, M_w_ = 114.06 g/mol, purity > 99.0%), (+)-α-Tocopherol (α-Tcp), 2,2-diphenyl-1-picrylhydrazyl (DPPH), and 1,4-dioxane (purity ≥ 99.0%) were all purchased from Sigma-Aldrich (Darmstadt, Germany), poly(vinyl alcohol) (PVA, M_w_ = 72,000 g/mol, 98% hydrolyzed) was acquired from Merck (Hohenbrunn, Germany), thymol (Thy, 2-isopropyl-5-methylphenol, C_10_H_14_O) was obtained from Alfa Aesar (Kandel, Germany), anhydrous 1-hexanol was purchased from Across-Organics (Geel, Belgium), monosodium phosphate (NaH_2_PO_4_ × 2H_2_O). Disodium phosphate (Na_2_H_2_PO_4_ × 7H_2_O) was procured from Chemical Company (Iasi, Romania) and ethanol (absolute, ≥99.8%) from Honeywell (Seelze, Germany). All chemicals were used as received without further purification.

### 4.2. Preparation of Cryogels by In Situ Entrapment of Thymol and α-Tocopherol

The cryogels were individually obtained by mixing proper ratios of PVA and PEBSA_Thy_α-Tcp complex solutions (Table 3), which were poured into molds and then subjected to three consecutive freeze–thaw cycles, respectively, freezing for 18 h at −20 °C followed by thawing for 8 h at 25 °C (ambient temperature) [13].

Briefly, PEBSA was synthesized as previously described [19] by a polycondensation procedure of EB macrolactone after ring-opening with SA. The new bioactive compound was obtained by the initial preparation of the PEBSA_Thy_α-Tcp complex produced by mixing PEBSA (0.066 g/mL in 1,4-dioxane) with different amounts of Thy and α-Tcp to obtain the desired PEBSA/Thy/α-Tcp mass ratio (either 1/1/1, 1/2/1, or 1/1/2 *w*/*w*/*w*). Then, the obtained complex was mixed with the PVA solution (4% *w*/*v*) in a volumetric ratio of 2/1. The synthesized samples were frozen with liquid nitrogen and lyophilized for 24 h at −55 °C (Alpha 1-2LD Plus, Martin Christ, Germany) for further characterization.

### 4.3. Characterization

#### 4.3.1. Morphological Analysis

The morphology in the cross-sections of the freeze-dried samples was observed by scanning electron microscopy (SEM Quanta 200, FEI Company, Hillsboro, OR, USA). The instrument operated with secondary electrons at 20 kV in low-vacuum mode, without any coating. Before analysis, the samples were fixed on aluminum stubs with double-adhesive carbon tape.

#### 4.3.2. Release Studies

To study the in vitro release behavior of the bioactive compounds, each sample was weighed (20 mg) and incubated in 10 mL of PBS, 0.01 M, at a constant temperature of 37 °C for 24 h. The release profiles of the bioactive substances were measured under different conditions using buffer solutions of pH 5.4 and 7.4 to simulate the pH of normal healthy skin and, respectively, the pH of injured skin. At predetermined time intervals, 2 mL of each sample was extracted and analyzed at 283 nm using a UV-VIS spectrophotometer (Jenway 6305, Stone, Staffordshire, United Kingdom). The cumulative release of Thy and α-Tcp was calculated based on the calibration curves determined at the same wavelengths.

#### 4.3.3. Antimicrobial Activity

The antimicrobial activity of the PVA/PEBSA_Thy_α-Tcp systems was determined using a disk diffusion assay [36,37] against three different reference strains: Gram-positive bacterial strain, *Staphylococcus aureus* ATCC25923 (*S. aureus*); Gram-negative bacterial strain, *Escherichia coli* ATCC25922 (*E. coli*); and fungal strain, *Candida albicans* ATCC10231 (*C. albicans*). All microorganisms were stored at −80 °C in 20% glycerol. The bacterial strains were refreshed on trypticase soy agar (TSA) at 37 °C and the yeast strain was refreshed on Sabouraud dextrose agar (SDA) at 37 °C. Microbial suspensions were prepared with these cultures in sterile solution to obtain turbidity optically comparable to that of 0.5 McFarland standards. Volumes of 0.1 mL from each inoculum were spread onto TSA/SDA plates, and then the sterilized samples of 10 mm and 25 mg each were added.

To evaluate the antimicrobial properties, the growth inhibition was measured under standard conditions after 24 h of incubation at 37 °C. All tests were carried out in triplicate for each sample. After incubation, the samples were analyzed with SCAN1200^®^, version 8.6.10.0 (Interscience, Saint Nom la Brétèche - FRANCE).

#### 4.3.4. Antioxidant Efficiency

The free radical scavenging activity of the bioactive compounds was evaluated by the 2,2-diphenyl-1-picrylhydrazyl (DPPH) assay according to the methodology described by Brand-Williams et al. [38]. Briefly, 3 mL of ethanol and 20 mg of the sample were added to 0.3 mL of DPPH stock solution 0.5 mM in absolute ethanol. The control solution was prepared by mixing ethanol (3 mL) and DPPH stock solution (0.3 mL). The reaction mixture was incubated in a dark place at room temperature for 30 min. The changes in color, from intense violet to light yellow, were recorded spectrophotometrically at 517 nm (Jenway 6305 UV–VIS Spectrophotometer, Stone, Staffordshire, UK). The percentage of DPPH radical scavenging activity was calculated by the following equation:

$$\%\;\mathrm{DPPH}\;\mathrm{radical}\;\mathrm{scavenging}\;\mathrm{activity}=\frac{A_{C}-{\;A}_{S}}{A_{C}}\times 100$$

where A_c_ is the absorbance of the control DPPH solution and A_S_ is the absorbance of the DPPH solution containing samples; the values reported for each sample represents the mean of three independent measurements.

#### 4.3.5. Statistical Analysis

All experimental data were performed in triplicate and the results were expressed as mean ± standard error of the mean. Statistical analysis was performed with XLSTAT Ecology version 2019.4.1 software [39].

## Figures and Tables

**Table 1:** A summary of controlled release studies of bioactive compounds from hydrogels.

Scaffold Material	Bioactive Compounds		Cumulative Bioactive Compound Release in Different Release Media	Reference
	Thy	α-Tcp		
Chitosan hydrogels	✓	-	~ 70% of Thy release in artificial saliva and ~ 45% of Thy release in phosphate-buffered saline (PBS) after 4 h; 100% of Thy release in almost 48 h	[24]
Sodium alginate/chitosan hydrogels	-	✓	37.9 ± 5.18% of α-Tcp release in simulated body fluid after 24 h; a sustained release of 77.2 ± 11.51% over 14 days	[25]
Sodium alginate/poly(2-ethyl-2-oxazoline) chitosan-coated semi-interpenetrating hydrogels	✓	-	78.1 ± 1.7% of Thy release in PBS after 25 days	[26]
Hydroxypropyl-β-cyclodextrin hydrogels	✓	-	73.4–98.9% of Thy release in PBS, 7.4 after 6 h	[27]
Sodium caseinate/gelatin nanocomposite hydrogel	✓	-	71% of thyme essential oil in PBS, 7.4 supplemented with 20% ethanolafter 72 h	[28]
PVA/pyrrolidone hydrogel	✓	-	70% of Thy release in ethanol solution after 5 days	[29]
Pluronic^®^ F-127/nanocellulose hydrogel	-	✓	100% of α-Tcp release in 8 days	[30]

**Table 2:** Antimicrobial activity (mm) of the tested samples against the reference strains.

Sample	Inhibition Zone (mm) *		
	*S. aureus*	*E. coli*	*C. albicans*
PVA_PEBSA_25/75__Thy_α-Tcp	22.30 ± 0.14	21.90 ± 0.99	38.55 ± 1.48
PVA_PEBSA_50/50__Thy_α-Tcp	21.10 ± 0.00	19.15 ± 1.06	32.25 ± 3.18
PVA_PEBSA_75/25__Thy_α-Tcp	25.90 ± 0.70	25.45 ± 3.46	34.65 ± 0.91
PVA_PEBSA_50/50__2xThy_α-Tcp	27.05 ± 0.63	28.40 ± 0.14	37.80 ± 0.28
PVA_PEBSA_50/50__Thy_2xα-Tcp	21.80 ± 3.25	19.20 ± 0.00	28.25 ± 0.77

* Data are represented as mean ± standard deviation from triplicate experiments.

**Table 3:** Samples name and bioactive compound preparation.

Sample	PVA/PEBSA Ratio	Composition for a Volume of 5 mL Sample			
		PVA (g)	PEBSA (g)	Thymol (g)	α-Tocopherol (g)
PVA_PEBSA_25/75__Thy_α-Tcp	2/1	0.132	0.066	0.066	0.066
PVA_PEBSA_50/50__Thy_α-Tcp				0.066	0.066
PVA_PEBSA_75/25__Thy_α-Tcp				0.066	0.066
PVA_PEBSA_50/50__2xThy_α-Tcp				0.132	0.066
PVA_PEBSA_50/50__Thy_2xα-Tcp				0.066	0.132

## References

[1] Enoch S., Leaper D.J. (2008). Basic Science of Wound Healing. Surgery.

[2] Velnar T., Bailey T., Smrkolj V. (2009). The Wound Healing Process: An Overview of the Cellular and Molecular Mechanisms. J. Int. Med. Res..

[3] Aguilar T.A.F., HernándezNavarro B.C., Pérez J.A.M., Aguilar T.A.F., HernándezNavarro B.C., Pérez J.A.M. (2016). Endogenous Antioxidants: A Review of Their Role in Oxidative Stress.

[4] Na Y., Woo J., Choi W.I., Lee J.H., Hong J., Sung D. (2021). α-Tocopherol-Loaded Reactive Oxygen Species-Scavenging Ferrocene Nanocapsules with High Antioxidant Efficacy for Wound Healing. Int. J. Pharm..

[5] Comino-Sanz I.M., López-Franco M.D., Castro B., Pancorbo-Hidalgo P.L. (2021). The Role of Antioxidants on Wound Healing: A Review of the Current Evidence. JCM.

[6] Roehrs M., Valentini J., Paniz C., Moro A., Charão M., Bulcão R., Freitas F., Brucker N., Duarte M., Leal M. (2011). The Relationships between Exogenous and Endogenous Antioxidants with the Lipid Profile and Oxidative Damage in Hemodialysis Patients. BMC Nephrol..

[7] Srivastava S., Phadke R.S., Govil G., Rao C.N.R. (1983). Fluidity, Permeability and Antioxidant Behaviour of Model Membranes Incorporated with α-Tocopherol and Vitamin E Acetate. Biochim. Et Biophys. Acta (BBA)-Biomembr..

[8] Bonferoni M.C., Riva F., Invernizzi A., Dellera E., Sandri G., Rossi S., Marrubini G., Bruni G., Vigani B., Caramella C. (2018). Alpha Tocopherol Loaded Chitosan Oleate Nanoemulsions for Wound Healing. Evaluation on Cell Lines and Ex Vivo Human Biopsies, and Stabilization in Spray Dried Trojan Microparticles. Eur. J. Pharm. Biopharm..

[9] Gonnet M., Lethuaut L., Boury F. (2010). New Trends in Encapsulation of Liposoluble Vitamins. J. Control. Release.

[10] Haleem A., Chen S.-Q., Ullah M., Siddiq M., He W.-D. (2021). Highly Porous Cryogels Loaded with Bimetallic Nanoparticles as an Efficient Antimicrobial Agent and Catalyst for Rapid Reduction of Water-Soluble Organic Contaminants. J. Environ. Chem. Eng..

[11] Ambreen J., Haleem A., Shah A.A., Mushtaq F., Siddiq M., Bhatti M.A., Shah Bukhari S.N.U., Chandio A.D., Mahdi W.A., Alshehri S. (2023). Facile Synthesis and Fabrication of NIPAM-Based Cryogels for Environmental Remediation. Gels.

[12] Wang M., Bai J., Shao K., Tang W., Zhao X., Lin D., Huang S., Chen C., Ding Z., Ye J. (2021). Poly(Vinyl Alcohol) Hydrogels: The Old and New Functional Materials. Int. J. Polym. Sci..

[13] Nita L.E., Crețu B.-E.-B., Șerban A.-M., Rusu A.G., Rosca I., Pamfil D., Chiriac A.P. (2023). New Cryogels Based on Poly (Vinyl Alcohol) and a Copolymacrolactone System. II. Antibacterial Properties of the Network Embedded with Thymol Bioactive Agent. React. Funct. Polym..

[14] Nagoor Meeran M.F., Javed H., Al Taee H., Azimullah S., Ojha S.K. (2017). Pharmacological Properties and Molecular Mechanisms of Thymol: Prospects for Its Therapeutic Potential and Pharmaceutical Development. Front. Pharmacol..

[15] Braga P.C., Dal Sasso M., Culici M., Bianchi T., Bordoni L., Marabini L. (2006). Anti-Inflammatory Activity of Thymol: Inhibitory Effect on the Release of Human Neutrophil Elastase. Pharmacology.

[16] Marchese A., Orhan I.E., Daglia M., Barbieri R., Di Lorenzo A., Nabavi S.F., Gortzi O., Izadi M., Nabavi S.M. (2016). Antibacterial and Antifungal Activities of Thymol: A Brief Review of the Literature. Food Chem..

[17] Escobar A., Pérez M., Romanelli G., Blustein G. (2020). Thymol Bioactivity: A Review Focusing on Practical Applications. Arab. J. Chem..

[18] Najafloo R., Behyari M., Imani R., Nour S. (2020). A Mini-Review of Thymol Incorporated Materials: Applications in Antibacterial Wound Dressing. J. Drug Deliv. Sci. Technol..

[19] Chiriac A.P., Rusu A.G., Nita L.E., Macsim A.-M., Tudorachi N., Rosca I., Stoica I., Tampu D., Aflori M., Doroftei F. (2021). Synthesis of Poly(Ethylene Brassylate-Co-Squaric Acid) as Potential Essential Oil Carrier. Pharmaceutics.

[20] Crețu B.-E.-B., Nita L.E., Șerban A.-M., Rusu A.G., Doroftei F., Chiriac A.P. (2022). New Cryogels Based on Poly(Vinyl Alcohol) and a Copolymacrolactone System: I-Synthesis and Characterization. Nanomaterials.

[21] Chiriac A.P., Asandulesa M., Stoica I., Tudorachi N., Rusu A.G., Nita L.E., Chiriac V.M., Timpu D. (2022). Comparative Study on the Properties of a Bio-Based Copolymacrolactone System. Polym. Test..

[22] Chiriac A.P., Stoleru E., Rosca I., Serban A., Nita L.E., Rusu A.G., Ghilan A., Macsim A.-M., Mititelu-Tartau L. (2022). Development of a New Polymer Network System Carrier of Essential Oils. Biomed. Pharmacother..

[23] Rusu A.G., Chiriac A.P., Nita L.E., Ghilan A., Rusu D., Simionescu N., Tartau L.M. (2022). Nanostructured Hyaluronic Acid-Based Hydrogels Encapsulating Synthetic/ Natural Hybrid Nanogels as Promising Wound Dressings. Biochem. Eng. J..

[24] Alvarez Echazú M.I., Olivetti C.E., Anesini C., Perez C.J., Alvarez G.S., Desimone M.F. (2017). Development and Evaluation of Thymol-Chitosan Hydrogels with Antimicrobial-Antioxidant Activity for Oral Local Delivery. Mater. Sci. Eng. C.

[25] Ehterami A., Salehi M., Farzamfar S., Samadian H., Vaez A., Ghorbani S., Ai J., Sahrapeyma H. (2019). Chitosan/Alginate Hydrogels Containing Alpha-Tocopherol for Wound Healing in Rat Model. J. Drug Deliv. Sci. Technol..

[26] Lavanya K., Balagangadharan K., Chandran S.V., Selvamurugan N. (2023). Chitosan-Coated and Thymol-Loaded Polymeric Semi-Interpenetrating Hydrogels: An Effective Platform for Bioactive Molecule Delivery and Bone Regeneration in Vivo. Biomater. Adv..

[27] Garg A., Ahmad J., Hassan M.Z. (2021). Inclusion Complex of Thymol and Hydroxypropyl-β-Cyclodextrin (HP-β-CD) in Polymeric Hydrogel for Topical Application: Physicochemical Characterization, Molecular Docking, and Stability Evaluation. J. Drug Deliv. Sci. Technol..

[28] Alsakhawy S.A., Baghdadi H.H., El-Shenawy M.A., Sabra S.A., El-Hosseiny L.S. (2022). Encapsulation of Thymus Vulgaris Essential Oil in Caseinate/Gelatin Nanocomposite Hydrogel: In Vitro Antibacterial Activity and in Vivo Wound Healing Potential. Int. J. Pharm..

[29] Malka E., Caspi A., Cohen R., Margel S. (2022). Fabrication and Characterization of Hydrogen Peroxide and Thymol-Loaded PVA/PVP Hydrogel Coatings as a Novel Anti-Mold Surface for Hay Protection. Polymers.

[30] Afrin Shefa A., Park M., Gwon J.-G., Lee B.-T. (2022). Alpha Tocopherol-Nanocellulose Loaded Alginate Membranes and Pluronic Hydrogels for Diabetic Wound Healing. Mater. Des..

[31] Jones E.M., Cochrane C.A., Percival S.L. (2015). The Effect of PH on the Extracellular Matrix and Biofilms. Adv. Wound Care.

[32] Darabian B., Bagheri H., Mohammadi S. (2020). Improvement in Mechanical Properties and Biodegradability of PLA Using Poly(Ethylene Glycol) and Triacetin for Antibacterial Wound Dressing Applications. Prog. Biomater..

[33] You S., Huang Y., Mao R., Xiang Y., Cai E., Chen Y., Shen J., Dong W., Qi X. (2022). Together Is Better: Poly(Tannic Acid) Nanorods Functionalized Polysaccharide Hydrogels for Diabetic Wound Healing. Ind. Crops Prod..

[34] Li Y., Fu R., Duan Z., Zhu C., Fan D. (2022). Adaptive Hydrogels Based on Nanozyme with Dual-Enhanced Triple Enzyme-Like Activities for Wound Disinfection and Mimicking Antioxidant Defense System. Adv. Healthc. Mater..

[35] Yang Y., Xu H., Li M., Li Z., Zhang H., Guo B., Zhang J. (2022). Antibacterial Conductive UV-Blocking Adhesion Hydrogel Dressing with Mild On-Demand Removability Accelerated Drug-Resistant Bacteria-Infected Wound Healing. ACS Appl. Mater. Interfaces.

[36] Bauer A.W., Perry D.M., Kirby W.M. (1959). Single-Disk Antibiotic-Sensitivity Testing of Staphylococci; an Analysis of Technique and Results. AMA Arch. Intern. Med..

[37] Clinical and Laboratory Standards Institute (CLSI) (2022). Performance Standards for Anti-Microbial Susceptibility Testing.

[38] Brand-Williams W., Cuvelier M.E., Berset C. (1995). Use of a Free Radical Method to Evaluate Antioxidant Activity. LWT-Food Sci. Technol..

[39] XLSTAT Statistical Software for Excel. https://www.xlstat.com/en/.

